# The Effect of Compositional Changes Due to Seasonal Variation on Milk Density and the Determination of Season-Based Density Conversion Factors for Use in the Dairy Industry

**DOI:** 10.3390/foods9081004

**Published:** 2020-07-27

**Authors:** Puneet Parmar, Nicolas Lopez-Villalobos, John T. Tobin, Eoin Murphy, Arleen McDonagh, Shane V. Crowley, Alan L. Kelly, Laurence Shalloo

**Affiliations:** 1 Livestock Systems Department, Teagasc Food Research Centre, Moorepark, Fermoy, Cork, Ireland; puneet.parmar@gmail.com (P.P.); arleen22@gmail.com (A.M.); 2 School of Food and Nutritional Sciences, University College Cork, Cork, Ireland; shane.crowley@ucc.ie (S.V.C.); a.kelly@ucc.ie (A.L.K.); 3 School of Agriculture and Environment, Massey University, Palmerston North 4442, New Zealand; N.Lopez-Villalobos@massey.ac.nz; 4 Food Chemistry and Technology Department, Teagasc Food Research Centre, Moorepark, Fermoy, Cork, Ireland; john.tobin@teagasc.ie (J.T.T.); eoin.murphy@teagasc.ie (E.M.)

**Keywords:** seasonal variation, raw milk, whole milk, composition, milk density, conversion

## Abstract

The objective of this study was to determine the effect of seasonal variation on milk composition and establish an algorithm to predict density based on milk composition to enable the calculation of season-based density conversion calculations. A total of 1035 raw whole milk samples were collected from morning and evening milking of 60 spring-calving individual cows of different genetic groups, namely Jersey, Elite HF (Holstein–Friesian) and National Average HF, once every two weeks for a period of 9 months (March–November, 2018). The average mean and standard deviation for milk compositional traits were 4.72 ± 1.30% fat, 3.85 ± 0.61% protein and 4.69 ± 0.30% lactose and density was estimated at 1.0308 ± 0.002 g/cm^3^. The density of the milk samples was evaluated using three methods: a portable density meter, DMA 35; a standard desktop version, DMA 4500M; and an Association of Official Agricultural Chemists (AOAC) method using 100-mL glass pycnometers. Statistical analysis using a linear mixed model showed a significant difference in density of milk samples (*p* < 0.05) across seasonal and compositional variations adjusted for the effects of days in milk, parity, the feeding treatment, the genetic group and the measurement technique. The mean density values and standard error of mean estimated for milk samples in each season, i.e., spring, summer and autumn were 1.0304 ± 0.00008 g/cm^3^, 1.0314 ± 0.00005 g/cm^3^ and 1.0309 ± 0.00007 g/cm^3^, respectively.

## 1. Introduction

Milk and dairy products are important components in the majority of western diets. The composition of milk significantly impacts the quality of final products, acceptability by consumers, and profitability of the dairy industry [1]. Over the past years, multiple studies have been performed to assess variations in the composition of milk. Several factors have been found to be directly or indirectly linked to the changes in milk composition [2,3,4,5]. Some of these factors include breed and genotype effects, changes in feeding systems, and the impact of seasonal changes and climatic conditions [6,7,8,9,10]. Climatic conditions may include high temperature variations, microclimate and cold weather conditions. High temperatures may induce heat stress in animals and heat stress has been observed for milk characteristics in Italy [11] and fatty acid composition in Swiss [12], Swedish [2] and Dutch milk [5].

Other factors linked to milk composition include lactation stage [13], animal health [14], herd management and farm and feed management practices [15,16]. The effect of processing on milk composition such as chemical composition, amino acids and fatty acid profile were studied in Ireland [9,17,18,19,20,21] and other parts of the world [22,23,24]. It has been reported that the availability and concentrations of different constituents of milk, such as fat and protein along with other physico-chemical properties, vary throughout a year [24,25]. This has been mainly attributed to the changes in feeding pattern and the stage of lactation [4]. When cows are grazed outdoors, changes in the feed are induced due to variable climatic conditions and growth stages of the grass that can introduce changes into the milk composition on a frequent basis. Change in the feed type and its effect on milk composition was studied [26] while significant compositional variations were observed when the diet was switched from silage-based to pasture-based and vice versa [27].

Significant variations in fat concentration, fatty acid profile and cheese yield in relation to feed patterns were reported in the past [8,9,28]. Similarly, alterations in feed leading to changes in milk composition have a significant effect on product quality [9,29]. Milk fat and protein content are the two main components that vary significantly due to seasonal variability in feed [30]. A study in the UK showed that the fat content in bovine milk collected between 2009–2013 decreased from January to July, followed by a sharp increase in August and September, remaining constant thereafter [25], while protein content declined steadily from November to April (3.35% to 3.23%), remained constant (April to July), and increased marginally thereafter [25].

Milk composition affects physical attributes like density and, thus, the basis of weight–volume calculations in the dairy processing industry. Changes in density are closely related to solids-non-fat content and fat content of milk [31], higher milk fat represents lower density and vice versa. The density of milk fluctuates between 1.025 to 1.035 g/cm^3^ [32] with seasonal changes throughout the year, resulting in higher densities in summer and lower in winter [24]. Density has also been noted to be dependent upon other factors such as temperature and processing conditions like agitation and homogenization [33,34].

The density of milk within a temperature range of 0–60 °C has been studied [35]; the density reduced from 1.0338 g/cm^3^ at 0.5 °C and to 1.0296 g/cm^3^ at 20 °C, while further decreasing with increasing temperature (1.0220 g/cm^3^ at 40 °C and 1.0132 g/cm^3^ at 60 °C). The physical state of fat globules becomes important at different temperatures, with crystallisation at lower temperatures (higher density) and melting of fat at higher temperatures (lower density) [36]. The impact of seasonal variation in milk composition profile has been assessed by various studies in the past, but its impact on milk density has not been studied extensively. Milk density is an important parameter in the dairy industry for estimating weight–volume relationships. In dairy processing, milk is supplied in volume (litres) while the final product mix is usually measured as mass/weight (kg), which may introduce variations in measurement. Current practice includes using an average single annual density factor to convert weight to volume; however, milk composition profile varies with different parameters, as stated earlier. Therefore, the use of a single density conversion factor for the weight–volume relationship in a processing environment is not representative of the seasonal changes in milk composition and may cause incorrect estimation of milk constituents (as it does not account for variations in composition observed over different seasons) highlighted in later sections.

The current study was designed to assess seasonal changes observed in raw milk composition by monitoring variations in individual milk constituents over a period of 9 months, covering spring, summer and autumn periods in Ireland. These seasonal changes in raw milk profile were then correlated with milk density to establish a density–composition relationship. The density–composition relationship helped to evaluate patterns of variation in density across different seasons and determine season-based density conversion factors which can be used by dairy processors to accurately estimate the yield of products and profitability of individual processors and the dairy industry as a whole.

## 2. Material and Methods

### 2.1. Experimental Design and Sample Collection

The experiment was carried out over a period of approximately 9 months from March 2018 to November 2018, divided into spring (March, April and May), summer (June, July, August) and autumn (September, October and November) seasons. Raw whole milk samples from spring-calved cows was collected from evening and morning milking from the Teagasc Research farm, Kilworth, Co. Cork (Latitude 50°07′ N, Longitude 08°16′ W). In a spring calving system, cows are calved close to the time when grass grows rapidly, allowing farmers to maximise production from grazed grass, subsequently positively impacting the profitability of their farm. Cows were selected based on their economic breeding index (EBI) (genetic merit) and the individual animal performance. The genetic groups assessed in this study included Jersey and Elite and National Average genetic merit Holstein–Friesian cows. All the cows (*n* = 60 total, 20 of each genetic group) included in the study were healthy and milked twice a day at 0700 and 1500 h.

Days in milk (DIM) was used as a parameter in the analysis for variation in milk density with season and stage of lactation. The spring calving period for the cows used in this study started at the end of January and continued until the third week of March. Spring season was classified for samples collected between March to May (DIM = 1–123), summer season for samples collected between June to August (DIM = 79–210) and autumn season for samples collected between September to November (DIM = 173–299), respectively.

The cows were also segregated into three groups, for each breed, based on feed. Between six and seven cows from each genetic group were selected based on EBI to be included for each diet pattern and were classified as control, high concentrate and low grass allowance groups [37]. The description of feed allowance is given below.

(a) High grass allowance: Stocking rate of 2.75 cows/ha, 250 kg N/year. Three kg of concentrate was offered per cow per day immediately post calving to supplement pasture availability in the spring for 12 weeks. Pasture was allocated in accordance with best management practice (approx. 4.5 cm post grazing residual). A grass only diet was offered in the autumn period for 12 weeks.

(b) High concentrate system: Stocking rate of 2.75 cows/ha. Concentrate (7 kg) was offered per cow per day immediately post-calving to supplement pasture availability in the spring for 12-weeks. Supplementation of 4 kg/day per cow was offered in the autumn period for 12 weeks.

(c) Low grass allowance: Similar to control with a lower post-grazing residual of 3.5–4.0 cm in spring and autumn.

A total of 1035 milk samples (combined morning + evening milk), approx. 150 mL each, were collected during this period and each of the samples were tested for compositional profile and whole milk density. The evening samples were collected once every two weeks and stored in a standard refrigerator at 4–5 °C overnight to prevent spoilage, while morning samples collected the next morning were then mixed with these to create a representative sample for analysis. The samples were proportionately mixed based on milk yield for the morning and evening milking to ensure that a representative sample was prepared, which was then properly agitated to ensure thorough mixing of constituents and to remove errors due to settling. Sampling requirements were in accordance with ISO 707:2008 (Milk and Milk Products: Guidance on sampling).

### 2.2. Sample Analysis

The following parameters were tested during the process: milk fat, protein and lactose content and raw milk density. A sample of approximately 30 mL was required for testing on the Dairyspec infrared manual FT model (Make-Bentley systems, Chaska, MN, USA) calibrated for raw whole milk compositional analysis. Milk density (measured at 20 °C, for all three equipment) was determined using three different pieces of equipment, i.e., DMA 35 portable density meter, DMA 4500 desktop density meter (Make-Anton Paar GmbH, Graz, Austria) and 100-mL calibrated glass pycnometers (Make-BRAND GMBH + CO KG, Wertheim,, Germany), following the procedure described by AOAC standard 925.22.

Before analysis, the density meters were calibrated using distilled water. The measured density of water on DMA 35 was 0.9974 g/cm^3^ and, for DMA 4500, it was 0.99826 g/cm^3^. The values fall under permissible limits of the theoretical value of 0.9982 g/cm^3^ for water at 20 °C. DMA 35 is commonly used for density measurement across industry due to its easier handling and manoeuvrability. DMA 35 works on the FTIR (Fourier transform infrared spectroscopy) principle of a hollow oscillating U-tube technology; the principle of operation is based on changing frequency of a hydrogen-filled hollow oscillator when filled with different liquids. The mass and density of the liquid changes the natural frequency of the oscillator due to overall change in mass of the oscillator when a liquid is added into the tube. The DMA 4500 also works on the similar principle of FTIR as described above. DMA 4500 has an operational range of temperature 0–100 °C and takes only 1–2 mL of sample for density measurement. The equipment is capable of automated cleansing and introduces immediate temperature equilibrium. The measurement principle and method of operation makes it robust and independent of manual interference, thus, reducing risk of errors in measurement. The sample was tested on the DMA 35 with approx. 1–2 mL sample drawn directly from the sample container, and density was noted from the display screen of the equipment. Syringes (2 mL) were used to inject the samples into the oscillating tubes of the DMA 4500 equipment, preventing the flow of air into the sample. Additional sample could be injected into the equipment if air bubbles were noticed on the display, which enabled optimization of the sample measurement to eliminate any errors.

The third method of measuring density was the AOAC 925.22 official method for determining the specific gravity of a liquid using pycnometer. The densities of liquids attained from the pycnometer method are obtained against water. In this method, firstly, an empty glass pycnometer was weighed and noted. The glass pycnometer was then filled with distilled water and wiped dry to remove any water molecules on the outer surface of the pycnometer. This filled weight was then measured and noted, after which the pycnometer was emptied completely. The pycnometer was then filled with liquid (milk) and the outer surface was wiped dry and weighed again. Excess liquid or water from the pycnometer was removed from the pycnometer through a capillary action of the pycnometer lid. The density of the liquid against water was measured using the formula

$$Density=\frac{WS-WE}{WW-WE}$$

where *WS* is the weight of the sample-filled pycnometer, *WE* is the weight of the empty pycnometer, and, *WW* is the weight of the water-filled pycnometer.

### 2.3. Statistical Analysis

The data for each sampling run were collected and collated for profile and density values for each season. The collected data were firstly analyzed to estimate the distribution of composition throughout the monitored period. Descriptive statistics (mean, standard deviation, minimum and maximum values) for density and milk compositional profile were determined using the MEANS procedure of Statistical Analysis Software (SAS) 9.4 (SAS Institute, Cary, NC, USA). Analyses of variance of the dependent variables (contents of fat, protein and lactose and density) were performed with a linear mixed model using the MIXED procedure of SAS 9.4 (SAS Institute, Cary, NC, USA). The model included the fixed effects of the genetic group, the feeding treatment, parity, the analytical approach for density measurement, days in milk with the linear and quadratic effect as the covariate and random effects of the cow and residual error.

A prediction model was developed using the linear mixed model for estimating density values considering the feeding treatment, the season, the measurement instrument, the genetic group, parity, the interaction between genetic group and the season, the linear effects of percentages of fat, protein and lactose, the linear and quadratic effects of days in milk, and random effects of the cow.

## 3. Results

A total of 1035 samples (combined morning + evening) were collected, and analyzed to obtain the descriptive statistics results shown in Table 1. The average fat content in milk samples was 4.72 ± 1.30%, and protein, casein, total solids and lactose contents were 3.85 ± 0.61%, 2.88 ± 0.58%, 14.02 ± 2.65% and 4.69 ± 0.30%, respectively, while average density for the study period was estimated at 1.0308 ± 0.0021 g/cm^3^. Table 1 also shows the somatic cell count (SCC), calculated as somatic cell score (SCS = log10 (SCC)), which is a marker for hygienic quality of milk samples. The somatic cell score (SCS) average was estimated at 4.66 ± 0.48, while the average somatic cell count was estimated at ~93,300 cells/mL. The somatic cell score calculated for the period of study had no significant impact on milk density found during analysis (*p* > 0.05). Table 2 shows the variations in the composition of milk constituents along with the standard error of the mean with fat contents; there was no significant difference between the seasons of spring (5.00 ± 0.14%) and autumn (5.13 ± 0.14%), while a significantly lower fat content (*p* < 0.05) was obtained in summer (4.71 ± 0.11%). On the other hand, protein content for each season was not significantly different (*p* > 0.05) (3.93 ± 0.05% protein in spring, 3.86 ± 0.04% protein in summer and 3.92 ± 0.05% protein in autumn) and lactose content varied significantly in autumn (*p* < 0.05) compared to the seasons of summer and spring (4.59 ± 0.26% in spring, 4.62 ± 0.17% in summer and 4.68 ± 0.31% in autumn). There was a significant difference in casein content in summer and spring season (*p* < 0.05), while no significant difference was found in casein content for autumn compared to spring and summer (3.00 ± 0.06% in spring, 2.91 ± 0.04% in summer, and 2.93 ± 0.05% in autumn). The total solids content with standard error of mean was significantly different (*p* < 0.05) for autumn when compared to spring and summer (13.95 ± 0.37% in spring, 13.68 ± 0.32% in summer, and 14.72 ± 0.37% in autumn). Descriptive statistics for the complete dataset showed that the minimum density was observed in April, at 1.0298 ± 0.0016 g/cm^3^, while maximum density was observed in the autumn period (November at 1.0316 ± 0.0022 g/cm^3^).

As shown in Table 3, the highest density value was obtained for the summer season (1.0314 ± 0.00005 g/cm^3^) while the lowest density value was estimated for the spring season (1.0304 ± 0.00008 g/cm^3^) and autumn had an intermediate density value of 1.0309 ± 0.00007 g/cm^3^. There were significant differences in density values for all the seasons (*p* < 0.05), with greatest difference being between spring and summer season (0.001 g/cm^3^). All the parameters, i.e., the season, the feeding treatment, the instrument, the genetic group of the animal, parity, the days in milk, and the days in milk squared as well as milk constituents, i.e., fat, lactose and protein, had a significant  effect on the variation in milk  density (*p* < 0.05), as also shown by the probability values estimated for the factors during analysis (Table 4). The interactive effect of genetic group and season was the only factor which was not significant (*p* > 0.05), while parity of the animal was also a significant factor and could be included as a parameter in the model. Further analysis of results from the linear mixed model procedure showed significant differences (*p* < 0.05) between measurement techniques (pycnometers and DMA4500, pycnometers and DMA35) but no significant difference between the results for DMA35 and DMA4500. Table 4 also shows the parameters of a linear model to predict milk density, including the season, the feeding treatment, the measurement instrument, the genetic group, parity, the interaction between the genetic group and the season, the linear effects of percentages of fat, protein, lactose, the linear and quadratic effects of days in milk, and random effects of the cow.

## 4. Discussion

### 4.1. The Effect of Seasonal Variation and Photoperiod on Milk Composition

The effect of seasonal variation and other factors on milk compositional profile has been extensively studied in the literature in the past [2,3,4,11,38,39]. However, the most important parameters that affect milk composition are diet/feed and the stage of lactation [4,29]. The lactation period significantly affected the milk composition, with late-lactation milk having higher fat and protein content as compared to mid-lactation [29]. The results of this study also align with [29], wherein the fat and protein contents were higher during the later phase of lactation, lowest in the spring period and highest in the autumn period. The density of milk has previously been shown to be dependent on fat and solids-non-fat (SNF) content in milk, and is normally measured at 20 °C [32]. The results from our study show the variation in milk density with season and compositional changes, where the density values in the summer season (lowest fat content) were highest and comparatively lower (1.0309 g/cm^3^) in the autumn samples (with higher fat content). Microbiological factors such as somatic cell count were not exclusively included in our analysis. However, somatic cell count (SCC) and somatic cell score (SCS) of milk samples were determined for the study period. The average somatic cell count over the period of study was ~93,000 cells/mL, while the average SCS was estimated at 4.66. In the literature, SCC has been shown to impact milk composition, especially the lactose content of milk due to decreased synthesis of lactose [2]. However, in our study, SCC was within acceptable limits and, thus, no significant impact of SCC was found on milk composition (*p* > 0.05). The total solids content was also higher in the autumn period compared to the summer and spring periods, but there was no significant variation between the summer and spring periods. This is in line with other studies in the UK and Ireland where the total solids content decreased during the January to April and July to August periods [20,24]. As stated earlier, milk yield and compositional characteristics are affected by the stage of lactation and diet. Milk density is dependent on milk fat and SNF content; therefore, the variation in total solids content also impacts milk density, increasing in the autumn season with increasing lactose and total solids contents of milk. The impact of variation in different constituents, i.e., protein and lactose, is also shown in Table 4 and was statistically significant. Fat content showed the highest variation when compared with protein and total solids, which is in line with the general observation that fat is the most sensitive to dietary changes [5,40]. The density results were determined for major constituents, i.e., milk, total protein and lactose, not segregated for casein (and whey) and/or total solids, to avoid multicollinearity errors in the analysis.

Diet plays a significant role in the variations observed in milk composition [2]. During the grazing season in Ireland, cows graze outdoors, and their diet is comprised mostly of fresh grass. Fatty acids form a significant component of milk fat and variation in fatty acid composition has been mainly attributed to the supply of fatty acids through diet and rumen microbial activity [5]. The main precursors of milk fat, i.e., acetic and butyric fatty acids—derived from rumen fermentation, can be affected by diet through changes in rumen fermentation or the addition of fats for direct absorption and inclusion into milk fat [2]. It has also been shown that the grass consumed by cows during grazing is less mature, and this less mature grass has lower levels of polyunsaturated fatty acids [41]. Oxidative losses in fatty acids due to the wilting and ensiling of grass have also been observed [42]. This reduces the amount of fatty acids from fresh grass and, thus, causes fluctuations in the fatty acid composition of milk, affecting the total fat content and milk density. Therefore, a combination of these factors and seasonal variation impacted the feed quality for grazing cows, which in turn affected the milk composition and milk density, respectively, as shown in results of this study.

Photoperiod is also known to have a significant impact on the milk production and compositional changes in milk. Photoperiod refers to the length of day or the period of daylight received by an organism [43], and the importance of photoperiod on the variations in milk composition has also been highlighted [44]. In dairy cattle, photoperiod influences a series of hormonal changes which affect the milk yield, composition and feed behaviour, among other parameters. Milk yield and dilution of fat and protein content have been reported to vary considerably with the increase in photoperiod or the length of the daylight period [44,45,46]. Photoperiod, as a factor, was not studied in this analysis but may contribute to the variation in milk composition and milk density and may thus require further analysis and exploration.

### 4.2. The Effect of Seasonal Variations on Milk Density, Mass Balances and Milk Payment Systems

It is evident from past research and the results of this study that seasonal variations introduce significant fluctuations in fat and protein content, increasing towards the autumn season. The variations in density values can be estimated using the model developed in this study. Variations in different parameters introduce differences in density values and, therefore, the use of a single density conversion factor is not representative of seasonal variations, including compositional changes, climatic conditions and feed practices.

The method of density analysis is also another important factor that can affect the accuracy of measurements. The results shown in this study indicate a significant impact of the measuring technique on the raw milk density for all the samples studied (Table 4, analytical method, *p* < 0.001). The differences in desntiy results between different analytical methods were observed. The pycnometer method was found to have statistically signficant differences with both DMA 35 and DMA 4500 (*p* < 0.001); however, DMA 35 and DMA 4500 results were not significantly different from each other (*p* > 0.05) over the period of study. DMA 35 is used in industry for quick analysis of density (Source: interactions with industry personnel), while DMA 4500 and pycnometer methods are comparatively time-consuming. The results of the pycnometer method were higher than the other two methods, and this may be attributed to different factors, such as accuracy and tolerance limits of the measuring equipment, foreign matter in samples like sediment and particulate matter, entrapped air and bubble formation, viscosity and homogenity of samples, and temperature and temperature history of samples. In this study, the analysis was carried out in a controlled environment using strong experimental protocols to remove errors or bias.

A mass balance may be defined as the consideration of the input, output and distribution of a product/ingredient between streams in a process. For a butter manufacture process, it may be presented as follows [47]:

Fat intake=Fat in products+losses+recycled fat

The use of a density factor is paramount in terms of a mass balance calculation that can help identify different loss-making points in a process, estimate losses in the fat conversion process and, subsequently, make important process-related and investment-related decisions. Milk payment systems across different regions follow the a multiple component pricing model (A + B - C system), where the value of protein (A) and fat (B) in kg supplied by the farmer to the processor are calculated and the cost of collection and processing (C) in cents per litre, related to the volume of milk supplied by the farmer, is deducted [47]. Milk volume is converted to weight using the density conversion factor by multiplying the volume collected in litres on each farm by the density factor to obtain the weight of milk in kg.

As stated earlier, the profile of milk in Ireland has considerably changed and a single density conversion factor is not representative of the variations in milk profile due to composition and seasonality. To put this in perspective, a hypothetical example is discussed here. The annual supply of milk in Ireland for the year 2019 was 7990 million L of milk [48] with the seasonal profile as supplied, corresponding to a peak milk supply of 13.4% in the month of May and trough of 2.2% in January. Milk distribution for the year 2019 varied between a maximum of 1072.2 million L in May, with the lowest supply observed in December (243.7 million L) and January (175.3 million L). Thus, using season-based density factors, milk weight was determined, giving a peak of 1105.33 million kg (using a density value of 1.0309 g/cm^3^) in May, while the minimum weight of milk was calculated for the December (251.38 million kg) and January (180.72 million kg) period using a density factor of 1.0314 g/cm^3^. Peak values of milk weight were obtained towards the end of spring and the beginning of the summer period when the milk supply was also at its highest (May–July). When an average density factor (1.0297 g/cm^3^, current industry standard) was used to calculate milk weight as compared to the density factors determined in this study, there was a total difference of 9.39 million kg/year in milk kg produced, with monthly differences as high as up to 1.3 million kg.

The model defined in this study can be a useful tool to predict the milk density value that can be used to estimate weight–volume calculations, based on different parameters such as the season, days in milk etc. Milk weight estimated using the predicted density may then be used to determine the fat and protein (in kg) available for processing. This variation in milk weight and constituents estimated from the use of new density factors will require appropriate planning. With proper planning and capacity appropriation, the processors can therefore have better operational control in terms of product mix and capacities, as well as a better understanding of their overall mass balance, while also presenting a more accurate financial picture by having the seasonal density factors calculated appropriately.

## 5. Conclusions

The density of milk is dependent upon seasonal variations observed in milk composition throughout the year. This is evident from the results of the present study, with density varying significantly with changes in the constituents’ content of the milk. Variations in the composition and ultimately density could be attributed to various factors, such as the stage of lactation, climatic conditions (including microclimatic pattern), the feeding pattern during the period of study, housing conditions in autumn and winter seasons, the genetic group, and temperature, amongst other parameters. Seasonal and annual factors for density conversion used in weight–volume relationships were determined, with an emphasis on usage of a periodic, rather than an average, conversion factor evident from the strength of linear regression models. The distribution of density and individual constituents of milk over the different seasons showed a similar trend, with higher fat and protein content observed in the autumn and winter seasons and the lowest content of these observed during summer. Monthly and season-based density factors were determined, which are relevant for milk-processing planning. Milk density is an important factor in milk processing to estimate the individual milk constituents (weight–volume calculations). The constituent contents thus calculated significantly influence the product portfolio, in conjunction with operating capacities and market demand. The use of season-based density factors, therefore, may improve upon the estimation of individual milk constituents, as shown from this study and, thus, it is vital for the processing industry to plan and control their product mix and operations more effectively. The estimation of new density factors may also enable improvements in the milk payment systems for the production and processing industry.

## Figures and Tables

**Table 1:** Descriptive statistics of milk composition, somatic cell score and density in milk in samples (*n* = 1035) collected from Jersey *(n* = 20) and Elite (*n* = 20) and National Average (*n* = 20) Holstein–Friesian cows over a period of 9 months (March–November 2018).

Trait	Mean	SD	Minimum	Maximum
Fat, %	4.72	1.30	2.14	14.86
Protein, %	3.85	0.61	1.76	5.95
Lactose, %	4.69	0.30	2.45	5.61
Casein, %	2.88	0.58	0.61	5.00
Total Solids, %	14.02	2.65	8.66	22.48
SCS (SCC × ‘000) ^1^	4.66 (93.3)	0.48 (3.35)	3.00 (1)	6.39 (2452)
Density, g/cm^3^	1.0308	0.0021	1.0153	1.0378

^1^ Somatic cell score (SCS) calculated as = log_10_(SCC), SCC = somatic cell count measured in ‘000 cells/mL.

**Table 2:** Least squares means and standard error of the mean (SEM) of milk composition in samples (*n* = 1035) collected from Jersey (*n* = 20) and Elite (*n* = 20) and National Average (*n* = 20) Holstein–Friesian cows over a period of 9 months (March–November 2018).

Trait	Season	Mean	SEM
Fat, %	Spring	5.00 ^a^	0.14
	Summer	4.71 ^b^	0.11
	Autumn	5.13 ^a^	0.14
Protein, %	Spring	3.93 ^a^	0.05
	Summer	3.86 ^a^	0.04
	Autumn	3.92 ^a^	0.05
Lactose, %	Spring	4.59 ^a^	0.26
	Summer	4.62 ^a^	0.17
	Autumn	4.68 ^b^	0.31
Total Solids, %	Spring	13.95 ^a^	0.37
	Summer	13.68 ^a^	0.32
	Autumn	14.72 ^b^	0.37
Casein, %	Spring	3.00 ^a^	0.06
	Summer	2.91 ^b^	0.04
	Autumn	2.93 ^a^	0.05

^a,b,c^ Means with different superscript within each milk component are significantly different (*p*-value < 0.05).

**Table 3:** Least squares means and standard error of the mean (SEM) of milk density in samples (*n* = 1035) collected from Jersey (*n* = 20) and Elite (*n* = 20) and National Average (*n* = 20) Holstein–Friesian cows over a period of 9 months (March–November 2018).

Season	Mean	SEM
Autumn	1.0309 ^b^	0.00007
Spring	1.0304 ^a^	0.00008
Summer	1.0314 ^c^	0.00005

^a,b,c^ Means with different superscript are significantly different (*p*-value < 0.05).

**Table 4:** Estimates of parameters and *p*-values of a linear model to predicted milk density, including the season, the feeding treatment, the measurement instrument, the genetic group, parity, the interaction between the genetic group and the season, the linear effects of percentages of fat, protein, lactose, the linear and quadratic effects of days in milk, and random effects of the cow, in Jersey (*n* = 20) and Elite (*n* = 20) and National Average (*n* = 20) Holstein–Friesian cows.

Effect	Genetic Group	FT	Season	Instrument	Parity	Estimate	*p*-Value
Intercept						1.00700	
FT							0.024
		HC				0.00012	
		HGA				9.26 × 10^−6^	
		LGA				0.00000	
Season							<0.0001
			Autumn			−0.00054	
			Spring			−0.00097	
			Summer			0.00000	
Instrument							<0.0001
				Pycnometer		0.00205	
				DMA35		−0.00006	
				DMA4500		0.00000	
Genetic Group							<0.0001
	Elite HF					0.00009	
	Jersey					0.00036	
	NA HF					0.00000	
Parity							0.0037
					1	0.00035	
					2	0.00032	
					3	0.00044	
					4	0.00041	
					5	0.00023	
					6	0.00053	
					8	0.00000	
Genetic group × season							0.5545
	Elite HF		Autumn			−0.00002	
	Elite HF		Spring			−0.00015	
	Elite HF		Summer			0.00000	
	Jersey		Autumn			−0.00003	
	Jersey		Spring			−0.00016	
	Jersey		Summer			0.00000	
	NA HF		Autumn			0.00000	
	NA HF		Spring			0.00000	
	NA HF		Summer			0.00000	
dim						−0.00002	<0.0001
dim * dim						6.713 × 10^−8^	<0.0001
Fat						−0.00066	<0.0001
Protein						0.00305	<0.0001
Lactose						0.00342	<0.0001

(Elite HF = Elite Holstein–Friesian, NA HF = National Average Holstein–Friesian; FT = feeding treatment, HC = high concentrate feeding, HGA = high grass allowance, LGA = low grass allowance; dim = days in milk).
